# Supplementary material for: Isolation and Characterization of Beauveria caledonica (Ascomycota: Hypocreales) Strains for Biological Control of Odoiporus longicollis Oliver (Coleoptera: Curculionidae)
Source: Microorganisms. 2025 Mar 28;13(4):782. doi: 10.3390/microorganisms13040782 (PMC12029869; doi:10.3390/microorganisms13040782)
Supplement: Supplementary file 1 [file microorganisms-13-00782-s001.zip › microorganisms-3391955-supplementary.pdf]

Supplementary materials

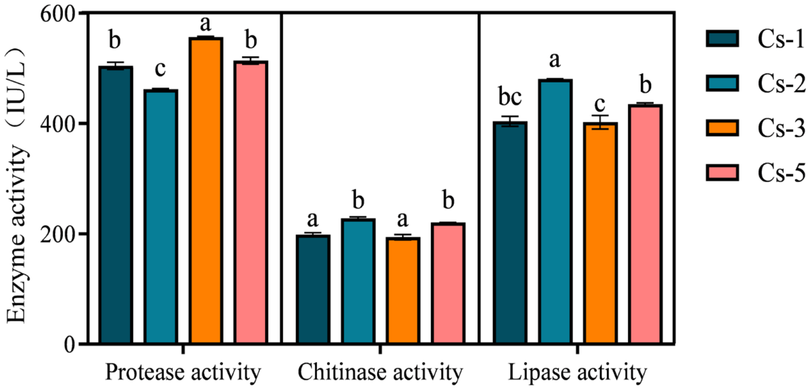

Figure S1. Enzyme activity of different strains.

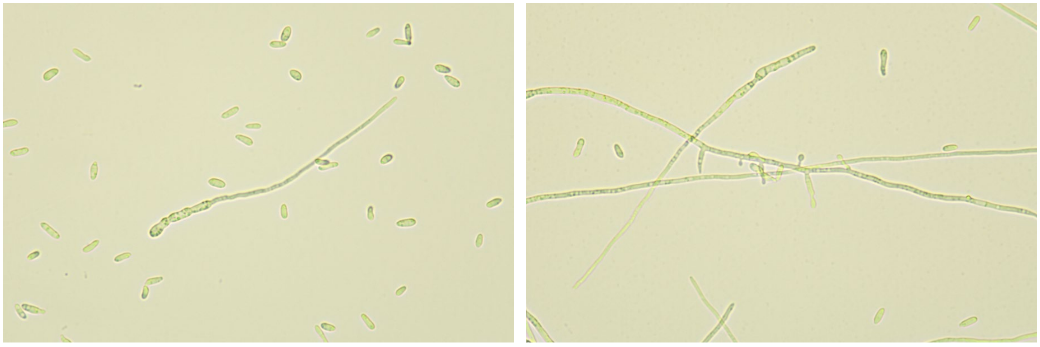

Figure S2. Morphological characteristics of *B. caledonica* under optical microscope.

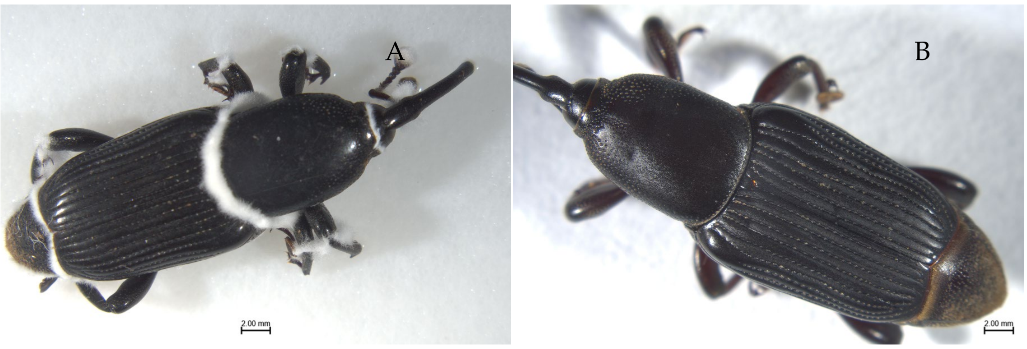

Figure S3. Morphological images of *O. longicollis* (Oliver) under stereomicroscope. (A): *O. longicollis* (Oliver) infected by *B. caledonica*; (B): Normal *O. longicollis* (Oliver).
